# Adverse Childhood Experiences in the First 1000 Days of Life and Dental Caries Experience From Age 31 to 61 Months

**DOI:** 10.1111/jphd.70012

**Published:** 2025-09-30

**Authors:** Noora Jawad, Ali Golkari, Eduardo Bernabé

**Affiliations:** ^1^ Institute of Dentistry Queen Mary University of London London UK

**Keywords:** ALSPAC, dental caries, domestic violence, early life stress, longitudinal studies

## Abstract

**Objective:**

To investigate the association between early life adverse childhood experiences (ACEs) and dental caries among young children.

**Methods:**

This study was a secondary analysis of longitudinal data from 927 children in the Children‐In‐Focus (CIF) sub‐study of the Avon Longitudinal Study of Parents and Children (ALSPAC). ACEs were assessed through questionnaires from mothers and partners, covering the gestation period (prenatal ACEs) and the first 2 years of life (ACEs during infancy). Dental caries was assessed clinically at ages 31, 43, and 61 months and reported using the dmft index. The association between early life ACEs and dental caries was evaluated in mixed effects Poisson models adjusting for covariates.

**Results:**

Overall, 342 (36.9%) and 441 (47.6%) children in the study sample were exposed to ACEs prenatally and during infancy, respectively. The mean dmft score was 0.03 ± 0.21 at age 31 months, increasing to 0.26 ± 0.94 at age 43 months and to 0.62 ± 1.51 at age 61 months. Neither exposure to ACEs prenatally (rate ratio: 1.11, 95% CI: 0.73–1.70) nor during infancy (1.08, 95% CI: 0.71–1.63) was associated with the dmft score, after adjustment for covariates. In addition, exposure to ACEs during both life periods was not associated with the dmft score (1.21, 95% CI: 0.74–1.98).

**Conclusion:**

This longitudinal study provided little support for the association between experience of ACEs during the first 1000 days of life and greater caries experience from age 31 to 61 months.

## 1 Introduction

The first 1000 days of life, from conception to the age of 2 years, are a critical period characterized by rapid growth and development [1]. Experiences and exposures during this period can influence developmental pathways and consequently shape an individual's life trajectory of health and disease [2, 3]. Identifying and addressing adversity in early life is essential for promoting healthy development and mitigating its short and long‐term negative impacts.

Adverse childhood experiences (ACEs) are distressing events, such as child maltreatment (physical, emotional, or sexual abuse, emotional neglect) and household dysfunction (parental separation, mental illness, partner violence, substance misuse, imprisonment), that occur in the immediate environment and will require genuine refitting [4, 5]. Exposure to ACEs generates chronic, excessive stress that exceeds a child's ability to cope, especially in the absence of supportive caregiving from adults [6, 7]. Such stress can alter gene expression, physiological processes, and neurocognitive and socioemotional development [8, 9, 10]. Adversity can begin prenatally, such as through maternal mental health illness or substance misuse, which may result in early harm to the offspring, including adverse pregnancy outcomes, developmental delays, social–emotional problems, and behavioral problems [11, 12]. In addition, the health effects of ACEs experienced prenatally and in early infancy can be observed from a very young age [12, 13, 14, 15, 16].

Despite the overwhelming evidence on the importance of the first 1000 days for people's health, very few dental studies have focused on this life stage. Contrarily, several reviews have evaluated the association of ACEs with early childhood caries [17], oral health in children and adolescents [18] and oral health in adults [19, 20]. However, the scoping review by Folayan, Schroth (17), which is the most relevant to the present study, found no clear evidence on the association of ACEs (*n* = 2 cross‐sectional studies) or the ACE domain of intimate partner violence (*n* = 4 cross‐sectional studies) with early childhood caries. Similarly, a recent 2‐year longitudinal study among 179 caries‐free 1–3‐year‐olds from New York, USA, showed that parent‐reported measures of psychological symptoms, alcohol abuse, and violence exposure were not associated with caries onset [21]. It remains to be determined whether the relationship between ACEs and oral health is apparent at very young ages. Longitudinal studies can provide valuable insight into this important area of research.

The aim of this study was to evaluate the association between early life ACEs (prenatally and during the first 2 years of life) and dental caries among young children. It was hypothesized that exposure to ACEs would be associated with greater caries experience.

## 2 Materials and Methods

### 2.1 Data Source

The Avon Longitudinal Study of Parents and Children (ALSPAC) is an ongoing cohort which recruited 14,541 expectant mothers from three health districts in Bristol, England, who were due to give birth between April 1st 1991 and December 31st 1992. Of them, 14,062 were born alive and 13,988 children survived to 1 year of age. ALSPAC has collected extensive data through questionnaires completed by mothers, partners, and children from pregnancy onward [22, 23].

The present study focused on a subset of participants from the ALSPAC study, specifically the Children‐In‐Focus (CIF) sub‐study. This group comprised around 10% of ALSPAC children born in the last 6 months of the recruitment phase (June to December 1992, *n* = 1432 ever attended). The selection process involved a pseudo‐random method based on the mother's birth date (even or odd day). The CIF sub‐study was designed to gather data through direct physical and psychological examinations, complementing the information collected via parental questionnaires in the main ALSPAC study. Children in the CIF sub‐study participated in research clinics at multiple early childhood time points, which corresponded to expected developmental milestones and growth phases. The CIF assessments took place at approximately 4, 8, 12, 18, 25, 31, 37, 43, 49, 61 months, of which those at 31, 43, and 61 months included dental examinations [24]. The response rate for CIF was consistently high, with about 80% attendance at each assessment period. CIF participants tended to have mothers who were older and more educated compared with the remaining ALSPAC cohort [23].

Ethical approval for the study was obtained from the ALSPAC Ethics and Law Committee and the Local Research Ethics Committees. The Children‐In‐Focus (CIF) study, whose data were used for this report, was approved by the Southmead Health Services (reference 48/89). Written informed consent from the parent/legal guardian of participants to participate was not directly obtained but inferred by completion of the questionnaire/participation in the survey.

### 2.2 Measurements

The study outcome was dental caries experience, which was determined from clinical examinations at ages 31, 43, and 61 months [24]. The first two examinations were conducted by trained dentists, and the third examination was conducted by two trained health professionals. The presence of cavitated lesions and existing restorations was determined visually using the British Association for the Study of Community Dentistry (BASCD) criteria [25]. The sum of decayed, missing, and filled deciduous teeth (dmft index) was calculated for each participating child at every examination. Examiners were trained through six tutorial sessions (16 h), followed by a one‐hour mock examination and replication on 30 children. The Kappa statistic for inter‐examiner reliability at the dmft level was 0.634.

The exposure was the experience of ACEs during the first 1000 days of life, covering the gestation period (prenatal ACEs) and the first 2 years of life (ACEs during infancy). Information on ACEs was extracted from the questionnaires completed by mothers and their partners at 12, 15, 18, and 32 weeks of gestation and at 8 weeks and 8, 18, and 21 months after birth. For inclusion, questions needed to match standard ACE definitions and be suitable for dichotomisation [26]. The list of items and their assessment time is shown in the Supporting Information. Prenatal ACEs included emotional abuse (*n* = 1 item), parental mental illness (*n* = 17), household conviction (*n* = 2), parental separation (*n* = 4), and substance misuse (*n* = 8). ACEs during infancy included emotional abuse (*n* = 9), parental mental illness (*n* = 20), parental conviction (*n* = 7), parental separation (*n* = 12), physical abuse (*n* = 9), substance misuse (*n* = 20), violence between parents (*n* = 5), and sexual abuse (*n* = 1). Based on participants' responses, binary indicators were created to indicate exposure to each ACE domain. These binary indicators were subsequently combined to determine the overall prevalence of ACEs prenatally and during infancy, respectively. In sensitivity analysis, the number of affected ACE domains was counted to generate ACE scores at each life stage. Scores ranged from 0 to 5 prenatally and from 0 to 8 during infancy, with higher scores indicating greater exposure to ACEs. Finally, information on exposure to ACEs in each life period was combined to generate four ACE trajectories from gestation to infancy, namely not exposed to ACEs at all (no‐no), not exposed prenatally but exposed during infancy (no‐yes), exposed prenatally but not during infancy (yes‐no), and exposed in both periods (yes‐yes).

Several additional variables were included in the analysis as covariates of the association between early life ACEs and childhood dental caries. They were child demographic factors (sex and ethnicity), maternal demographic factors (age at delivery and marital status), and family socioeconomic status (maternal education and parental social class). Social class was determined based on the respondent's occupation during pregnancy and grouped into six categories: professional (I); managerial and technical (II); skilled non‐manual (IIINM); skilled manual (IIIM); partly skilled (IV); and unskilled (V). We used the highest social class of the mother or partner (if available) and collapsed the six original categories into four (I, II, IIINM, and IIIM‐V) due to small numbers in some categories.

### 2.3 Data Analysis

All analysis was carried out in Stata 18 MP (StataCorp, College Station, TX). The significance level was set at 0.05. We first compared the characteristics of participants in the study sample with those excluded from the analysis due to missing data using the Chi‐square test. Then, the prevalence of prenatal ACEs and ACEs during infancy was compared according to covariates using the Chi‐square test for unordered groups and Cochran‐Armitage test for linear trends for ordered groups. We also compared dmft scores at every wave (ages 31, 43, and 61 months) according to exposure to prenatal ACEs, ACEs during infancy, and ACEs trajectories. These cross‐sectional comparisons were made in crude Poisson regression models.

Mixed‐effects Poisson regression was used to model the change in dmft score from age 31 to 61 months, with the three repeated caries assessments (level 1) nested within children (level 2). Mixed‐effects models make use of all available outcome data (using full‐information maximum likelihood estimation to handle data missingness), effectively managing instances of missing or unevenly distributed observations over time. They also account for the correlations that occur among repeated measurements from the same individual [27, 28]. Child age was used as the time indicator and modeled flexibly using a categorical indicator (age 31 = 1, age 43 = 2 and age 61 = 3). The intercept and slope for child age were treated as random effects to characterize individual differences in baseline dmft score and its rate of change over time, respectively. All other model predictors were estimated as fixed effects. The covariance matrix was estimated with no restrictions (unstructured). The association between exposure to prenatal ACEs and dmft score from age 31 to 61 months was evaluated in two sequential models. Model 1 was adjusted for time, child demographic factors, mother sociodemographic factors, and family socioeconomic position. Model 2 was further adjusted for ACEs during infancy. The estimates from these models represent the effect of exposure to prenatal ACEs on dmft score at age 31 months that will continue unchanged until age 61 months (i.e., parallel lines for children exposed and unexposed to ACEs during gestation). The same two models were fitted to test the association between ACEs during infancy and dmft score over time. Finally, the association between ACE trajectories and dmft score was evaluated in a model adjusted for time, child demographic factors, mother sociodemographic factors, and family socioeconomic position.

## 3 Results

Of the 1432 children that were recruited in the CIF sub‐study, 1031, 1027, and 917 were clinically examined for dental caries at age 31, 43, and 61 months, respectively. Of the 1031 children who participated in the first caries examination, 104 were excluded because of missing data on relevant variables (maternal education = 65, child ethnicity = 44, parental social class = 44, marital status = 25, ACEs = 9, and maternal age = 4), leaving 927 children for inclusion in the analysis. Of them, 674, 182, and 71 children contributed three, two, and one waves of caries data, respectively. There were no differences in demographic factors and exposure to ACEs between children included and excluded from the analysis. However, children in the study sample were more likely to have older and more educated mothers, and parents who were married and from higher social class (Table 1).

**TABLE 1:** Characteristics of the study sample and comparison with those excluded from the analysis due to missing data.

	Excluded (*n* = 104)		Study sample (*n* = 927)		*p* value^a^
	*n*	%	*n*	%	
*Child sex*					0.261
Male	62	59.6	499	53.8	
Female	42	40.4	428	46.2	
*Child ethnicity*					0.394
White	57	92.0	899	97.0	
Non‐white	< 5	8.0	28	3.0	
*Maternal age*					< 0.001
< 24 years	35	35.0	103	11.1	
25–34 years	53	53.0	721	77.8	
35–44 years	12	12.0	103	11.1	
*Maternal education*					< 0.001
Below high school	18	42.8	172	18.6	
High school	19	45.2	609	65.7	
Above high school	< 5	11.9	146	15.8	
*Parental social class*					< 0.001
I	< 5	6.3	140	15.1	
II	18	22.7	444	47.9	
IIINM	22	27.8	221	23.8	
IIIMV	34	43.0	122	13.2	
*Marital status*					< 0.001
Married	42	53.2	771	83.2	
Formerly married	9	11.4	43	4.6	
Single	28	35.4	113	12.2	
*Prenatal ACEs*					0.095
No	54	54.6	585	63.1	
Yes	45	45.5	342	36.9	
*ACEs during infancy*					0.400
No	48	48.0	486	52.4	
Yes	52	52.0	441	47.6	
*ACEs trajectories*					0.277
No‐No	31	32.6	390	42.1	
No‐Yes	22	23.2	195	21.0	
Yes‐No	14	14.7	96	10.4	
Yes‐Yes	28	29.5	246	26.5	

^a^ Chi‐square test was used for comparison.

Overall, 342 (36.9%) and 441 (47.6%) children in the study sample were exposed to ACEs prenatally and during infancy, respectively. Exposure to ACEs prenatally and during infancy was more prevalent among children of single and formerly married women. Exposure to prenatal ACEs was more common among children of younger mothers and those of parents with lower social class, whereas exposure to ACEs during infancy was more common among children of less educated mothers (Table 2).

**TABLE 2:** Adverse childhood experiences (ACEs) by child, maternal and family‐related covariates (*n* = 927).

	Prenatal ACEs		ACEs during infancy	
	*n*	%	*n*	%
*All children*	342	36.9	441	47.6
*Child sex*				
Male	182	36.5	245	49.1
Female	160	37.4	196	45.8
*p value* ^a^	*0.775*		*0.315*	
*Child ethnicity*				
White	332	36.9	429	47.7
Non‐white	10	35.7	12	42.9
*p value* ^a^	*0.896*		*0.612*	
*Maternal age at delivery*				
< 24 years	50	48.5	59	57.3
25–34 years	257	35.6	333	46.2
35–44 years	35	34.0	49	47.6
*p value for trend* ^a^	*0.030*		*0.163*	
*Maternal education*				
Below high school	71	41.3	96	55.8
High school	220	36.1	282	46.3
Above high school	51	34.9	63	43.2
*p value for trend* ^a^	*0.226*		*0.020*	
*Parental social class*				
I	45	32.1	55	39.3
II	162	36.5	221	49.8
IIINM	79	35.8	105	47.5
IIIMV	56	45.9	60	49.2
*p value for trend* ^a^	*0.046*		*0.256*	
*Marital status*				
Married	261	33.9	336	43.6
Formerly married	28	65.1	34	79.1
Single	53	46.9	71	62.8
*p value* ^a^	*< 0.001*		*< 0.001*	

^a^ Chi‐square test and Cochran‐Armitage test for linear trends were used to compare unordered and ordered groups, respectively.

The mean dmft score was 0.03 ± 0.21 at age 31 months, increasing to 0.26 ± 0.94 at age 43 months and to 0.62 ± 1.51 at age 61 months. In crude analyses (Table 3), children exposed to prenatal ACEs had a higher mean dmft score at age 61 months (0.73 ± 1.66) than unexposed children (0.57 ± 1.42, *p* = 0.009), but not at younger ages. No difference in mean dmft score was found between children exposed and unexposed to ACEs during infancy. Children exposed to ACEs both prenatally and during infancy had a higher mean dmft score (0.78 ± 1.77) than those unexposed in both periods (0.59 ± 1.59, *p* = 0.015) and those unexposed prenatally but exposed during infancy (0.52 ± 1.25, *p* = 0.004).

**TABLE 3:** Dental caries experience (dmft score) at ages 31, 43 and 61 months, by different indicators of exposure to adverse childhood experiences (ACEs).

	Age 31 months (*n* = 927)		Age 43 months (*n* = 809)		Age 61 months (*n* = 722)	
	Mean	(SD)	Mean	(SD)	Mean	(SD)
*Prenatal ACEs*						
No	0.03	(0.20)	0.25	(0.83)	0.57	(1.42)
Yes	0.03	(0.23)	0.27	(1.06)	0.73	(1.66)
*p value* ^*a*^	*0.793*		*0.326*		*0.009*	
*ACEs during infancy*						
No	0.04	(0.24)	0.27	(0.86)	0.60	(1.47)
Yes	0.02	(0.18)	0.34	(1.20)	0.66	(1.56)
*p value* ^*a*^	*0.050*		*0.481*		*0.288*	
*ACEs trajectories*						
No‐No	0.04	(0.23)	0.24	(0.83)	0.59	(1.59)
No‐Yes	0.04	(0.25)	0.25	(0.84)	0.61	(1.39)
Yes‐No	0.01	(0.07)	0.25	(0.71)	0.52	(1.25)
Yes‐Yes	0.03	(0.23)	0.29	(1.27)	0.78	(1.77)
*p value* ^*a*^	*0.211*		*0.721*		*0.018*	

^a^ Poisson regression was used to compare dmft scores according to ACEs groups.

The results from adjusted mixed‐effects models suggested a trend toward increased caries experience among children with early life ACEs, although all confidence intervals included the null value (Table 4). Children exposed to ACEs prenatally (rate ratio [RR]: 1.14, 95% CI: 0.76–1.70) and during infancy (RR: 1.12, 95% CI: 0.75–1.66) tended to have higher dmft scores between ages 31 and 61 months than their corresponding counterparts (Model 1). These estimates were somewhat attenuated after mutual adjustment (Model 2); children exposed to ACEs prenatally (RR: 1.11, 95% CI: 0.73–1.70) and during infancy (RR: 1.08, 95% CI: 0.71–1.63) tended to have higher dmft scores than unexposed children. In sensitivity analysis, neither the ACE score prenatally (RR: 1.12, 95% CI: 0.81–1.55) nor during infancy (RR: 1.02, 95% CI: 0.81–1.28) was associated with the dmft score. Finally, the analysis of ACE trajectories showed that children exposed to ACEs both prenatally and during infancy tended to have higher dmft scores (RR: 1.21, 95% CI: 0.74–1.98) than those unexposed in both periods.

**TABLE 4:** Models for the association between adverse childhood experiences (ACEs) and dental caries experience (dmft score) from age 31 to 61 months (2458 observations in 927 children).

	Model 1			Model 2		
	RR	[95% CI]	*p* value	RR	[95% CI]	*p* value
*Prenatal ACEs*						
No	1.00	[Reference]		1.00	[Reference]	
Yes	1.14	[0.76, 1.71]	0.521	1.11	[0.73, 1.70]	0.627
*ACEs during infancy*						
No	1.00	[Reference]		1.00	[Reference]	
Yes	1.12	[0.75, 1.65]	0.581	1.08	[0.71, 1.63]	0.721
*ACEs trajectories*						
No‐No	1.00	[Reference]				
No‐Yes	1.01	[0.60, 1.69]	0.981			
Yes‐No	0.99	[0.51, 1.92]	0.982			
Yes‐Yes	1.21	[0.74, 1.98]	0.434			

*Note:* Mixed‐effects Poisson regression model was fitted with repeated dmft scores (level 1) nested within children (level 2). Rate ratios (RR) were thus reported. Model 1 was adjusted for child sex and ethnicity, maternal age at delivery, marital status and education, and parental social class. Model 2 was additionally adjusted for the other indicator of exposure to ACEs.

## 4 Discussion

This longitudinal study found no association between ACEs in the first 1000 days of life and dental caries experience among young children. Neither exposure to ACEs prenatally nor during infancy was associated with caries experience from ages 31 to 61 months. Furthermore, exposure to ACEs during both life periods was not associated with caries experience.

Some explanations for this finding can be put forward. Our sample size could have played a role in explaining the absence of an association between ACEs and early childhood caries. A power analysis revealed that the study had 80% power to detect a difference in dmft of at least 0.25 teeth between children exposed and unexposed to ACEs, assuming the same mean dmft for unexposed children (0.57), the same standard deviations for the dmft in exposed and unexposed children (1.42 and 1.66), and the same ratio of exposed to unexposed children (585/342 = 1.71) as observed in the present study. However, the actual difference in dmft between the two groups was smaller (0.14 vs. the required 0.25). To detect the observed difference of 0.14 teeth, a sample of 2376 children (with 877 exposed to ACEs) would have been necessary; an impractical requirement for a longitudinal study.

Another explanation is the low levels of dental caries observed in our sample, which reduced variability in the dmft score and limited our ability to detect the hypothesized association. It is possible that an association between ACEs and early childhood caries might have been identified in a higher‐risk group. Furthermore, given evidence that exposure to ACEs is inversely associated with healthcare utilization during childhood [29, 30], it is possible that affected children were less likely to participate in the CIF's clinical examinations, including dental inspections. This may lead to an underestimation of ACE prevalence and potentially bias the associations between ACE and dental caries toward the null [26, 31].

It is also possible that an association between ACEs and early childhood caries does not exist. Our findings agree with those from a scoping review [17] and the only previous longitudinal study [21]. Notably, our findings indicated that the association was only modest. After adjusting for sociodemographic factors, children exposed to ACEs had around 8%–11% greater caries experience than those not exposed. These findings suggest that even with a larger sample, the association would likely remain of limited importance. They also point to the role of other early life factors, such as family socioeconomic circumstances, which play a stronger role in determining early childhood caries.

Of course, this does not mean that ACEs are unimportant for oral health. It simply indicates that exposure to ACEs during the first 1,000 days of life may not be relevant to early childhood caries. It may be too early in life to observe any detrimental effects on children's oral health. Longer‐term studies are needed to determine whether early ACEs influence oral health outcomes at later ages. ACEs do not occur in isolation [5]. They tend to cluster concurrently (multiple adversities coexisting) as well as longitudinally (repeated exposure to the same set of adversities) [4]. Our findings on the trajectories of ACEs appear to support this argument on cumulative exposure, as children continuously exposed to ACEs from gestation to age 2 years tended to have greater caries experience compared with those unexposed during both periods, whereas children exposed to ACEs in only one of these periods (either prenatally or during infancy) did not differ markedly from those unexposed throughout.

Although the present study did not find any association between ACEs and early childhood caries, this cannot be entirely ruled out at the present time. ACEs are a significant public health problem affecting people's health, including oral health at later stages in life [18, 19, 20]. The diverse impacts of ACEs imply that multidisciplinary approaches, integrating medical, social, and psychological services, are needed to address the complex health needs of individuals with ACEs. Health care providers, including dentists, could help identify vulnerable families or individuals in primary care settings. There is a need to increase awareness and training among dental teams to provide more sensitive and comprehensive care that considers the psychosocial background of patients. As for research, further longitudinal studies in similar and other age groups as well as in alternative populations are needed.

Strengths of this study included the use of prospectively collected data on ACEs and dental caries, the relatively large sample size, and control for relevant covariates. However, this study also has some limitations. Firstly, around 10% of children were excluded from the analysis because they did not have complete information on the variables chosen for analysis. While the observed relationships between variables remain valid, differences between analyzed and excluded groups limit the generalizability of findings to the broader study population. Secondly, some readers may question the relevance of data collected in the 1990s. However, the prevalence of untreated caries has remained unchanged over the past three decades [32] and recent national estimates of ACE prevalence closely mirror those reported in ALSPAC [33]. Furthermore, as almost all existing research in this area has been cross‐sectional [17], our longitudinal findings remain highly pertinent and provide valuable insights for current public health discussions. Thirdly, the moderate reliability during caries examinations (Kappa = 0.63) could have increased measurement error and affected our ability to identify some associations. Fourthly, although information on ACEs was collected prospectively, it was based on parental (mostly maternal) self‐reports, which are always prone to information bias (recall and social desirability biases). We cannot rule out the possibility that some ACEs were not reported in full. Finally, we could not evaluate intermediate factors of the association between ACEs and dental caries, such as dental behaviors. However, it must be said that this was beyond the focus of our study.

To conclude, this longitudinal study provides little support for the association between exposure to ACEs during the first 1000 days of life and greater caries experience among young children.

## Conflicts of Interest

The authors declare no conflicts of interest.

## Supporting information


**Data S1:** Supporting Information.

## Data Availability

The ALSPAC website contains details of all the data that is available through a fully searchable data dictionary and variable search tool (http://www.bristol.ac.uk/alspac/researchers/our‐data/).
