# Supplementary material for: Adverse Childhood Experiences in the First 1000 Days of Life and Dental Caries Experience From Age 31 to 61 Months
Source: J Public Health Dent. 2025 Sep 30;85(4):457–63. doi: 10.1111/jphd.70012 (PMC12689280; doi:10.1111/jphd.70012)
Supplement: Supplementary file 1 — Data S1: Supporting Information. [file JPHD-85-457-s001.docx]

**List of ALSPAC questionnaire items used to define Adverse Childhood Experiences (ACEs)**

| **Variable name** | **ACEs domain** | **Question** | **Response options** | **Binary indicator** | **Age at data collection** | **Life period** |
| --- | --- | --- | --- | --- | --- | --- |
| B608 | emotional abuse | Partner emotionally cruel since pregnancy | 1=Yes affected a lot  2=Yes fairly affected  3=Yes mildly affected  4=Yes but not effect at all  5=No didn’t happen | No: 5  Yes: 1 to 4 | 18-week gestation | Prenatal |
| B370 | parental mental illness | Edinburgh post-natal depression score | Score from 0 to 30 | No: Score 0 to 10  Yes: Score 11 to 30 | 18-week gestation | Prenatal |
| C600 | parental mental illness | Edinburgh post-natal depression score | Score from 0 to 30 | No: Score 0 to 10  Yes: Score 11 to 30 | 32-week gestation | Prenatal |
| pb260 | parental mental illness | Edinburgh post-natal depression score | Score from 0 to 30 | No: Score 0 to 10  Yes: Score 11 to 30 | 18 weeks gestation | Prenatal |
| b106 | parental mental illness | Medication for anxiety this pregnancy | 1=Yes, in 1-3 months  2=Yes, 4 months to now  3=Yes, both time periods  4=Not at all | Yes: 1 to 3  No: 4 | 18-week gestation | Prenatal |
| b107 | parental mental illness | Medication for anxiety in first 3 months | 1=Yes  2=No | Yes: 1  No: 0 | 18-week gestation | Prenatal |
| b122 | parental mental illness | Medication for depression this pregnancy | 1=Yes, in 1-3 months  2=Yes, 4 months to now  3=Yes, both time periods  4=Not at all | Yes: 1 to 3  No: 4 | 18-week gestation | Prenatal |
| b123 | parental mental illness | Medication for depression in 1st 3 months | 1=Yes  2=No | Yes: 1  No: 0 | 18-week gestation | Prenatal |
| b597 | parental mental illness | Attempted suicide since pregnancy | 1=Yes, affected a lot  2=Yes, mildly affected  3=Yes, but no effect at all  4=No, it didn’t happen | Yes: 1 to 3  No: 4 | 18-week gestation | Prenatal |
| c093 | parental mental illness | medications for anxiety in last 3 months | 1=Yes  2=No | Yes: 1  No: 0 | 32-week gestation | Prenatal |
| c101 | parental mental illness | medications for depression in last 3 months | 1=Yes  2=No | Yes: 1  No: 0 | 32-week gestation | Prenatal |
| d152 | parental mental illness | Had bulimia | 1=Yes, recently  2=Yes, in past  3=No, never | Yes: 1 to 2  No: 3 | 15-week gestation | Prenatal |
| d169 | parental mental illness | Had schizophrenia | 1=Yes, recently  2=Yes, in past  3=No, never | Yes: 1 to 2  No: 3 | 15-week gestation | Prenatal |
| d170 | parental mental illness | Had anorexia nervosa | 1=Yes, recently  2=Yes, in past  3=No, never | Yes: 1 to 2  No: 3 | 15-week gestation | Prenatal |
| pa172 | parental mental illness | Had bulimia | 1=Yes, recently  2=Yes, in past  3=No, never | Yes: 1 to 2  No: 3 | 12-week gestation | Prenatal |
| pa189 | parental mental illness | Had schizophrenia | Yes= recently  Yes= in past  No=never | Yes: 1 to 2  No: 3 | 12-week gestation | Prenatal |
| pa190 | parental mental illness | Had anorexia nervosa | 1=Yes, recently  2=Yes, in past  3=No, never | Yes: 1 to 2  No: 3 | 12-week gestation | Prenatal |
| pb187 | parental mental illness | Attempted suicide since partner’s pregnancy | 1=Yes, affected a lot  2=Yes, fairly affected  3=Yes, mildly affected  4=Yes, but no effect at all  5=No, it didn’t happen | Yes: 1 to 4  No: 5 | 18-week gestation | Prenatal |
| b598 | parental conviction | Convicted of an offence since pregnancy | 1=Yes, affected a lot  2=Yes, fairly affected  3=Yes, mildly affected  4=Yes, but no effect at all  5=No, it didn’t happen | Yes: 1 to 4  No: 5 | 18-week gestation | Prenatal |
| pb188a | parental conviction | Convicted of offence since partner’s pregnancy,Yes/No | 1=Yes  2=No | Yes: 1  No: 0 | 18-week gestation | Prenatal |
| b578 | parental separation | Divorced since pregnancy | 1=Yes, affected a lot  2=Yes, fairly affected  3=Yes, mildly affected  4=Yes, but no effect at all  5=No, it didn’t happen | Yes: 1 to 4  No: 5 | 18-week gestation | Prenatal |
| b587 | parental separation | Separated since pregnancy | 1=Yes, affected a lot  2=Yes, fairly affected  3=Yes, mildly affected  4=Yes, but no effect at all  5=No, it didn’t happen | Yes: 1 to 4  No: 5 | 18-week gestation | Prenatal |
| pb168 | parental separation | Divorced since partner’s pregnancy | 1=Yes, affected a lot  2=Yes, fairly affected  3=Yes, mildly affected  4=Yes, but no effect at all  5=No, it didn’t happen | Yes: 1 to 4  No: 5 | 18-week gestation | Prenatal |
| pb177 | parental separation | Separated since partner’s pregnancy | 1=Yes, affected a lot  2=Yes, fairly affected  3=Yes, mildly affected  4=Yes, but no effect at all  5=No, it didn’t happen | Yes: 1 to 4  No: 5 | 18-week gestation | Prenatal |
| b701 | substance misuse | Smoked cannabis in 1-3 months of pregnancy | 1=Yes, everyday  2=Yes, 2-4 times a week  3=Yes, once per week  4=Yes, <once per week  5=No, not at all | Yes: 1 to 4  No: 5 | 18-week gestation | Prenatal |
| b702 | substance misuse | Smoked cannabis after 3 months of pregnancy | 1=Yes, everyday  2=Yes, 2-4 times a week  3=Yes, once per week  4=Yes, <once per week  5=No, not at all | Yes: 1 to 4  No: 5 | 18-week gestation | Prenatal |
| b714 | substance misuse | Hard drugs | 1=Yes  2=No | Yes: 1  No: 0 | 18-week gestation | Prenatal |
| d167 | substance misuse | Had drug addiction | 1=Yes, recently  2=Yes, in past  3=No, never | Yes: 1 to 2  No: 3 | 15-week gestation | Prenatal |
| d168 | substance misuse | Had alcoholism | 1=Yes, recently  2=Yes, in past  3=No, never | Yes: 1 to 2  No: 3 | 15-week gestation | Prenatal |
| pa187 | substance misuse | Had drug addiction | 1=Yes, recently  2=Yes, in past  3=No, never | Yes: 1 to 2  No: 3 | 12-week gestation | Prenatal |
| pa188 | substance misuse | Had alcoholism | 1=Yes, recently  2=Yes, in past  3=No, never | Yes: 1 to 2  No: 3 | 12-week gestation | Prenatal |
| pb098 | substance misuse | Hard drugs | 1=Yes  2=No | Yes: 1  No: 0 | 18-week gestation | Prenatal |
| f257 | emotional abuse | Partner emotionally cruel to their child after the child born | 1=Yes, much affected  2=Yes, moderately affected  3=Yes, mildly affected  4=Yes, but no effect  5=No, it did not happen | Yes: 1 to 4  No: 5 | 8 months | Infancy |
| f258 | emotional abuse | Mother emotionally cruel to their child after the child born | 1=Yes, much affected  2=Yes, moderately affected  3=Yes, mildly affected  4=Yes, but no effect  5=No, it did not happen | Yes: 1 to 4  No: 5 | 8 months | Infancy |
| pd257 | emotional abuse | Partner emotionally cruel to children | 1=Yes, affected a lot  2=Yes, moderately affected  3=Yes, mildly affected  4=Yes, but no effect  5=No, it did not happen | Yes: 1 to 4  No: 5 | 8 months | Infancy |
| pd258 | emotional abuse | Emotionally cruel to Children | 1=Yes, affected a lot  2=Yes, moderately affected  3=Yes, mildly affected  4=Yes, but no effect  5=No, it did not happen | Yes: 1 to 4  No: 5 | 8 months | Infancy |
| pc236 | emotional abuse | Partner emotionally cruel to child | 1=Yes, affected a lot  2=Yes, moderately affected  3=Yes, mildly affected  4=Yes, but no effect  5=No, it did not happen | Yes: 1 to 4  No: 5 | 8 weeks | Infancy |
| g337 | emotional abuse | Partner emotionally cruel to children after children 8 months | 1=Yes, big effect  2=Yes, some effect  3=Yes, mild effect  4=Yes, but no effect  5=No, it did not happen | Yes: 1 to 4  No: 5 | 1 year 9 months | Infancy |
| g338 | emotional abuse | Mother emotionally cruel to children after children 18 months | 1=Yes, big effect  2=Yes, some effect  3=Yes, mild effect  4=Yes, but no effect  5=No, it did not happen | Yes: 1 to 4  No: 5 | 1 year 9 months | Infancy |
| pe337 | emotional abuse | Partner emotionally cruel to child | 1=Yes, affected a lot  2=Yes, moderately affected  3=Yes, mildly affected  4=Yes, but no effect  5=No, it did not happen | Yes: 1 to 4  No: 5 | 1 year 9 months | Infancy |
| pe338 | emotional abuse | Self emotionally cruel to child | 1=Yes, affected a lot  2=Yes, moderately affected  3=Yes, mildly affected  4=Yes, but no effect  5=No, it did not happen | Yes: 1 to 4  No: 5 | 1 year 9 months | Infancy |
| e326 | parental mental illness | frequency of anti-depressant use since birth | 1=Yes, almost daily  2=Yes, sometimes  3=No, not at all | Yes: 1 to 2  No: 3 | 8 weeks | Infancy |
| f063 | parental mental illness | Anti-depressant use since child born | 1=Yes, daily  2=Yes, often  3=Yes, sometimes  4=No, not at all | Yes: 1 to 3  No: 4 | 8 months | Infancy |
| f248 | parental mental illness | Attempted suicide after child born | 1=Yes, much affected  2=Yes, moderately affected  3=Yes, mildly affected  4=Yes, but no effect  5=No, it did not happen | Yes: 1 to 4  No: 5 | 8 months | Infancy |
| f526 | parental mental illness | Partner had schizophrenia after child born | 1=Yes, saw the doctor  2=Yes, but didn’t see the doctor  3=No=never | Yes: 1 to 2  No: 3 | 8 months | Infancy |
| pd063 | parental mental illness | Used Pills for depression since baby born | 1=Yes, every day  2=Yes, often  3=Yes, sometimes  4=No, not at all | Yes: 1 to 3  No: 4 | 8 months | Infancy |
| pd248 | parental mental illness | Attempted suicide since baby born | 1=Yes, affected a lot  2=Yes, mildly affected  3=Yes, but did not affect  4=No, it did not happen | Yes: 1  Yes: 3 to 4  No: 5 | 8 months | Infancy |
| e391 | parental mental illness | Edinburgh post-natal depression score | Score from 0 to 28 | No: score 0 to 10  Yes: score 11 to 28 | 8 weeks | Infancy |
| pc102 | parental mental illness | Edinburgh post-natal depression score | Score from 0 to 27 | No: score 0 to 10  Yes: score 11 to 27 | 8 weeks | Infancy |
| e427 | parental mental illness | Attempted suicide since mid-pregnancy | 1=Yes, affected a lot  2=Yes, moderately affected  3=Yes, mildly affected  4=Yes, but no effect  5=No, it did not happen | Yes: 1 to 4  No: 5 | 8 weeks | Infancy |
| pc227 | parental mental illness | Attempted suicide since mid-pregnancy | 1=Yes, affected a lot  2=Yes, moderately affected  3=Yes, mildly affected  4=Yes, but no effect  5=No, it did not happen | Yes: 1 to 4  No: 5 | 8 weeks | Infancy |
| f200 | parental mental illness | Edinburgh post-natal depression score | Score from 0 to 29 | No: score 0 to 10  Yes: score 11 to 29 | 8 months | Infancy |
| pd200 | parental mental illness | Edinburgh post-natal depression score | Score from 0 to 24 | No: score 0 to 10  Yes: score 11 to 24 | 8 months | Infancy |
| g049 | parental mental illness | Mum had depression pills after the child 8 months | 1=Yes, every day  2=Yes, often  3=Yes, sometimes  4=No, not at all | Yes: 1 to 3  No: 4 | 1 year 9 months | Infancy |
| g328 | parental mental illness | Mum attempted suicide after the child 8 months | 1=Yes, big effect  2=Yes, some effect  3=Yes, mild effect  4=Yes, but no effect  5=No, it did not happen | Yes: 1 to 4  No: 5 | 1 year 9 months | Infancy |
| g612 | parental mental illness | H3l: Whether partner had schizophrenia and accessed a doctor: G file | 1=Yes, saw doctor  2=Yes, but didn’t see doctor  3=No, not at all | Yes: 1 to 2  No: 3 | 1 year 9 months | Infancy |
| pe020 | parental mental illness | Anxiety since child after 8 Months | 1=Yes, consulted doctor  2=Yes, but did not consult doctor  3=No, it did not happen | Yes: 1 to 2  No: 3 | 1 year 9 months | Infancy |
| pe064 | parental mental illness | Taken antidepressants when child after 8 months | 1=Yes, every day  2=Yes, often  3=Yes, sometimes  4=Yes, rarely  5=No, never | Yes: 1 to 4  No: 5 | 1 year 9 months | Infancy |
| g290 | parental mental illness | Edinburgh post-natal depression score | Score from 0 to 30 | No: score 0 to 10  Yes: score 11 to 30 | 1 year 9 months | Infancy |
| pe290 | parental mental illness | Edinburgh post-natal depression score | Score from 0 to 27 | No: score 0 to 10  Yes: score 11 to 27 | 1 year 9 months | Infancy |
| pe328 | parental mental illness | Attempted Suicide | 1=Yes, affected a lot  2=Yes, moderately affected  3=Yes, mildly affected  4=Yes, but no effect  5=No, it did not happen | Yes: 1 to 4  No: 5 | 1 year 9 months | Infancy |
| e428 | parental conviction | Convicted since mid-pregnancy | 1=Yes, affected a lot  2=Yes, moderately affected  3=Yes, mildly affected  4=Yes, but no effect  5=No, it did not happen | Yes: 1 to 4  No: 5 | 8 weeks | Infancy |
| pc228a | parental conviction | Convicted since mid-pregnancy,Yes/No | 1=Yes  2=No | Yes: 1  No: 0 | 8 weeks | Infancy |
| f249a | parental conviction | Court conviction | 1=Yes  2=No | Yes: 1  No: 0 | 8 months | Infancy |
| pd249a | parental conviction | Convicted of offence since baby born | 1=Yes  2=No | Yes: 1  No: 0 | 8 months | Infancy |
| g329 | parental conviction | Mother convicted of offence after child is 8 months | 1=Yes, big effect  2=Yes, some effect  3=Yes, mild effect  4=Yes, but No effect  5=No, it did not happen | Yes: 1 to 4  No: 5 | 1 year 9 months | Infancy |
| g329a | parental conviction | Mother convicted of offence after child is 8 months | 1=Yes  2=No | Yes: 1  No: 0 | 1 year 9 months | Infancy |
| pe329a | parental conviction | Convicted of offenceYes/No | 1=Yes  2=No | Yes: 1  No: 0 | 1 year 9 months | Infancy |
| f228 | parental separation | Divorce after child born | 1=Yes, much affected  2=Yes, moderately affected  3=Yes, mildly affected  4=Yes, but no effect  5=No, it did not happen | Yes: 1 to 4  No: 5 | 8 months | Infancy |
| f237 | parental separation | Separation from partner after the child was born | 1=Yes, much affected  2=Yes, moderately affected  3=Yes, mildly affected  4=Yes, but no effect  5=No, it did not happen | Yes: 1 to 4  No: 5 | 8 months | Infancy |
| pd228 | parental separation | Divorced since baby born | 1=Yes, affected a lot  2=Yes, moderately affected  3=Yes, mildly affected  4=Yes, but no effect  5=No, it did not happen | Yes: 1 to 4  No: 5 | 8 months | Infancy |
| pd237 | parental separation | Separated since baby born | 1=Yes, affected a lot  2=Yes, moderately affected  3=Yes, mildly affected  4=Yes, but no effect  5=No, it did not happen | Yes: 1 to 4  No: 5 | 8 months | Infancy |
| e408 | parental separation | Divorced since mid-pregnancy | 1=Yes, affected a lot  2=Yes, moderately affected  3=Yes, mildly affected  4=Yes, but no effect  5=No, it did not happen | Yes: 1 to 4  No: 5 | 8 weeks | Infancy |
| e417 | parental separation | Separated since mid-pregnancy | 1=Yes, affected a lot  2=Yes, moderately affected  3=Yes, mildly affected  4=Yes, but no effect  5=No, it did not happen | Yes: 1 to 4  No: 5 | 8 weeks | Infancy |
| pc208 | parental separation | Divorced since mid-pregnancy | 1=Yes, affected a lot  2=Yes, moderately affected  3=Yes, mildly affected  4=Yes, but no effect  5=No, it did not happen | Yes: 1 to 4  No: 5 | 8 weeks | Infancy |
| pc217 | parental separation | You & partner separated since mid-pregnancy | 1=Yes, affected a lot  2=Yes, moderately affected  3=Yes, mildly affected  4=Yes, but no effect  5=No, it did not happen | Yes: 1 to 4  No: 5 | 8 weeks | Infancy |
| g308 | parental separation | Mother divorced after child is 8 months | 1=Yes, big effect  2=Yes, some effect  3=Yes, mild effect  4=Yes, but no effect  5=No, it did not happen | Yes: 1 to 4  No: 5 | 1 year 9 months | Infancy |
| g317 | parental separation | Mum and partner separated after child is 8 months | 1=Yes, big effect  2=Yes, some effect  3=Yes, mild effect  4=Yes, but no effect  5=No, it did not happen | Yes: 1 to 4  No: 5 | 1 year 9 months | Infancy |
| pe308 | parental separation | Divorced | 1=Yes, affected lots  2=Yes, Mildly Affected  3=Yes but did not affect  4=No, it did not happen | Yes: 1  Yes: 3 to 4  No: 5 | 1 year 9 months | Infancy |
| pe317 | parental separation | Separated from partner | 1=Yes, affected a lot  2=Yes, moderately affected  3=Yes, mildly affected  4=Yes, but no effect  5=No, it did not happen | Yes: 1 to 4  No: 5 | 1 year 9 months | Infancy |
| f246 | physical abuse | Partner physically cruel to child after child was born | 1=Yes, affected a lot  2=Yes, moderately affected  3=Yes, mildly affected  4=Yes, but no effect  5=No, it did not happen | Yes: 1 to 4  No: 5 | 8 months | Infancy |
| f247 | physical abuse | Mother physically cruel to child after child was born | 1=Yes, much affected  2=Yes, moderately affected  3=Yes, mildly affected  4=Yes, but no effect  5=No, it did not happen | Yes: 1 to 4  No: 5 | 8 months | Infancy |
| pc226 | physical abuse | Partner physical-cruel to child since mid-pregnancy | 1=Yes, affected a lot  2=Yes, moderately affected  3=Yes, mildly affected  4=Yes, but no effect  5=No, it did not happen | Yes: 1 to 4  No: 5 | 8 weeks | Infancy |
| pd246 | physical abuse | Partner physically cruel to children | 1=Yes, affected a lot  2=Yes, moderately affected  3=Yes, mildly affected  4=Yes, but no effect  5=No, it did not happen | Yes: 1 to 4  No: 5 | 8 months | Infancy |
| pd247 | physical abuse | Self physically cruel to children | 1=Yes, affected a lot  2=Yes, moderately affected  3=Yes, mildly affected  4=Yes, but no effect  5=No, it did not happen | Yes: 1 to 4  No: 5 | 8 months | Infancy |
| g326 | physical abuse | Partner physically cruel to children after child is 8 months | 1=Yes, big effect  2=Yes, some effect  3=Yes, mild effect  4=Yes, but no effect  5=No, it did not happen | Yes: 1 to 4  No: 5 | 1 year 9 months | Infancy |
| g327 | physical abuse | Mother physically cruel to children after child is 8 months | 1=Yes, big effect  2=Yes, some effect  3=Yes, mild effect  4=Yes, but no effect  5=No, it did not happen | Yes: 1 to 4  No: 5 | 1 year 9 months | Infancy |
| pe326 | physical abuse | Partner physically cruel to child | 1=Yes, affected a lot  2=Yes, moderately affected  3=Yes, mildly affected  4=Yes, but no effect  5=No, it did not happen | Yes: 1 to 4  No: 5 | 1 year 9 months | Infancy |
| pe327 | physical abuse | Self physically cruel to child | 1=Yes, affected a lot  2=Yes, moderately affected  3=Yes, mildly affected  4=Yes, but no effect  5=No, it did not happen | Yes: 1 to 4  No: 5 | 1 year 9 months | Infancy |
| kd505a | sexual abuse | Child sexually abused after 6 months | 1=Yes, child very upset  2=Yes, child quite upset  3=Yes, child a bit upset  4=No, it did not happen | Yes: 1 to 3  No: 5 | 18 months | Infancy |
| e190 | substance misuse | frequency of Ganja use in last 2 months of pregnancy | 1=Yes, daily  2=Yes, 2-4 per week  3=Yes, once per week  4=Yes, <once per week  5=No, not at all | Yes: 1 to 4  No: 5 | 8 weeks | Infancy |
| e203 | substance misuse | Hard drug in last 2 months | 1=Yes  2=No | Yes: 1  No: 0 | 8 weeks | Infancy |
| e192 | substance misuse | Frequency of Ganja use since birth | 1=Yes, daily  2=Yes, 2-4 per week  3=Yes, once per week  4=Yes, <once per week  5=No, not at all | Yes: 1 to 4  No: 5 | 8 weeks | Infancy |
| e213 | substance misuse | Hard drug use since delivery | 1=Yes  2=No | Yes: 1  No: 0 | 8 weeks | Infancy |
| pc266 | substance misuse | Frequency cannabis smoked since birth | 1=Yes, daily  2=Yes, 2-4 per week  3=Yes, once per week  4=Yes, <once per week  5=No, not at all | Yes: 1 to 4  No: 5 | 8 weeks | Infancy |
| f061 | substance misuse | Cannabis use since child born | 1=Yes, daily  2=Yes, often  3=Yes, sometimes  4=No, not at all | Yes: 1 to 3  No: 4 | 8 months | Infancy |
| f067 | substance misuse | Amphetamine use since child born | 1=Yes, daily  2=Yes, often  3=Yes, sometimes  4=No, not at all | Yes: 1 to 3  No: 4 | 8 months | Infancy |
| f069 | substance misuse | Opiate or cocaine use since child born | 1=Yes, daily  2=Yes, often  3=Yes, sometimes  4=No, not at all | Yes: 1 to 3  No: 4 | 8 months | Infancy |
| f527 | substance misuse | Partner had alcoholism after child was born | 1=Yes, saw doctor  2=Yes, but did not see doctor  3=No, never | Yes: 1 to 2  No: 3 | 8 months | Infancy |
| pd061 | substance misuse | Used Cannabis/Marijuana since baby born | 1=Yes, every day  2=Yes, often  3=Yes, sometimes  4=No, not at all | Yes: 1 to 3  No: 4 | 8 months | Infancy |
| pd066 | substance misuse | Used Amphetamines since baby born | 1=Yes, often  2=Yes, sometimes  3=No, not at all | Yes: 2 to 3  No: 4 | 8 months | Infancy |
| pd067 | substance misuse | Used Heroin, Cocaine since baby born | 1=Yes, every day  2=Yes, often  3=Yes, sometimes  4=No, not at all | Yes: 1 to 3  No: 4 | 8 months | Infancy |
| pc276 | substance misuse | Hard drugs | 1=Yes  2=No | Yes: 1  No: 0 | 8 weeks | Infancy |
| g047 | substance misuse | Mother had cannabis after child was 8 months | 1=Yes, every day  2=Yes, often  3=Yes, sometimes  4=No, not at all | Yes: 1 to 3  No: 4 | 1 year 9 months | Infancy |
| g053 | substance misuse | Mother had amphetamines after child was 8 months | 1=Yes, every day  2=Yes, often  3=Yes, sometimes  4=No, not at all | Yes: 1 to 3  No: 4 | 1 year 9 months | Infancy |
| g056 | substance misuse | Mother had heroin, meth, or cocaine after child was 8 months | 1=Yes, every day  2=Yes, often  3=Yes, sometimes  4=No, not at all | Yes: 1  Yes: 3  No: 4 | 1 year 9 months | Infancy |
| g613 | substance misuse | H3m: Whether partner had drink (alcohol) problem and accessed a doctor: G file | 1=Yes, saw doctor  2=Yes, but did not see doctor  3=No, not at all | Yes: 1 to 2  No:3 | 1 year 9 months | Infancy |
| pe062 | substance misuse | Taken Cannabis Since child after 8 months | 1=Yes, every day  2=Yes, often  3=Yes, sometimes  4=Yes, rarely  5=No, never | Yes: 1 to 4  No: 5 | 1 year 9 months | Infancy |
| pe067 | substance misuse | Taken Amphetamines since child after 8 months | 1=Yes, every day  2=Yes, often  3=Yes, sometimes  4=Yes, rarely  5=No, never | Yes: 1 to 4  No: 5 | 1 year 9 months | Infancy |
| pe069 | substance misuse | Taken Heroin/Cocaine child after 8 months | 1=Yes, every day  2=Yes, often  3=Yes, sometimes  4=Yes, rarely  5=No, never | Yes: 1 to 4  No: 5 | 1 year 9 months | Infancy |
| f242 | family violence | Physically hurt by partner after child was born | 1=Yes, much affected  2=Yes, moderately affected  3=Yes, mildly affected  4=Yes, but no effect  5=No, it= did not happen | Yes: 1 to 4  No: 5 | 8 months | Infancy |
| pd242 | family violence | Partner physically cruel since baby born | 1=Yes, affected a lot  2=Yes, moderately affected  3=Yes, mildly affected  4=Yes, but it did not affect  5=No, it did not happen | Yes: 1 to 4  No: 5 | 8 months | Infancy |
| pc222 | family violence | Partner physically hurt you since mid-pregnancy | 1=Yes, affected a lot  2=Yes, moderately affected  3=Yes, mildly affected  4=Yes, but it did not affect  5=No, it did not happen | Yes: 1 to 4  No: 5 | 8 weeks | Infancy |
| g322 | family violence | Partner physically cruel to mum after child 8 months | 1=Yes, big effect  2=Yes, some effect  3=Yes, mild effect  4=Yes, but no effect  5=No, it did not happen | Yes: 1 to 4  No: 5 | 1 year 9 months | Infancy |
| pe322 | family violence | Partner physically cruel | 1=Yes, affected a lot  2=Yes, moderately affected  3=Yes, mildly affected  4=Yes, but it did not affect  5=No, it did not happen | Yes: 1 to 4  No: 5 | 1 year 9 months | Infancy |
